# Missing in Action: Reports of Interdisciplinary Integration in Canadian Palliative Care

**DOI:** 10.3390/curroncol28040235

**Published:** 2021-07-16

**Authors:** Maggie C. Robinson, Maryam Qureshi, Aynharan Sinnarajah, Srini Chary, Janet M. de Groot, Andrea Feldstain

**Affiliations:** 1 Tom Baker Cancer Centre, Alberta Health Services, Calgary, AB T2N 4N2, Canada; Maggie.robinson@tcd.ie (M.C.R.); janet.degroot@albertahealthservices.ca (J.M.d.G.); 2 Werklund School of Education, University of Calgary, Calgary, AB T2N 1N4, Canada; mquresh@ucalgary.ca; 3 Department of Medicine, Queen’s University, Kingston, ON K7L 3J7, Canada; asinnarajah@lh.ca; 4 Department of Medicine, Lakeridge Health, Ajax, ON L1S 2J4, Canada; 5 Cumming School of Medicine, University of Calgary, Calgary, AB T2N 4N1, Canada; 6 Palliative Medicine, Queen’s University, Kingston, ON K7L 3J7, Canada; sc260@queensu.ca

**Keywords:** palliative care, psychosocial, interdisciplinary, healthcare, integration

## Abstract

Palliative care has an interdisciplinary tradition and Canada is a leader in its research and practice. Yet even in Canada, a full interdisciplinary complement is often lacking, with psychosocial presence ranging from 0–67.4% depending on the discipline and region. We sought to examine the most notable gaps in care from the perspective of Canadian palliative professionals. Canadian directors of palliative care programs were surveyed with respect to interdisciplinary integration. Participants responded in writing or by phone interview. We operationalized reports of interdisciplinary professions as either “present” or “under/not-represented”. The Vaismoradi, Turunen, and Bondas’ procedure was used for content analysis. Our 14 participants consisted of physicians (85.7%), nurses (14.3%), and a social worker (7.1%) from Ontario (35.7%), British Columbia (14.3%), Alberta (14.3%), Quebec (14.3%), Nova Scotia (14.3%), and New Brunswick (7.1%). Psychology and social work were equally and most frequently reported as “under/not represented” (5/14, each). All participants reported the presence of medical professionals (physicians and nurses) and these groups were not reported as under/not represented. Spiritual care and others (e.g., rehabilitation and volunteers) were infrequently reported as “under/not represented”. Qualitative themes included Commonly Represented Disciplines, Quality of Multidisciplinary Collaboration, Commonly Under-Represented Disciplines, and Special Concern: Psychosocial Care. Similar to previous reports, we found that (1) psychology was under-represented yet highly valued and (2) despite social work’s relative high presence in care, our participants reported a higher need for more. These finding highlight those psychosocial gaps in care are most frequently noted by palliative care professionals, especially psychology and social work. We speculate on barriers and enablers to addressing this need.

## 1. Introduction

Palliative care is a unique branch of medicine. It focuses on quality-of-life rather than cures and overall wellbeing in addition to physical illness. It was one of the first areas in medicine to recognize that quality-of-life comprises biological, psychological, social, and spiritual domains. It was developed by interdisciplinary clinicians and maintains interdisciplinary theory to address the needs of patients and their families [1,2,3]. Having a complete team to address these needs bolsters holistic care planning and integrates all aspects of care. Spiritual, social, and emotional factors can be addressed by psychologists, social workers, psychiatrists, and spiritual care clinicians (e.g., chaplains). Even though palliative care physicians and nurses have training in therapeutic conversations, it is unlikely that they can meet all of the psychosocial care needs for all of their patients, especially for those with higher levels of distress [4].

Canada has been qualified as a worldwide leader in palliative care [5]. Accordingly, Canadians are at the forefront of developing evidence-based psychosocial interventions for this patient population [6]. Despite the interdisciplinary tradition, development of targeted psychosocial interventions, and the clear literature on the importance of addressing psychosocial suffering [7,8,9], Canadian and international palliative care services often experience disparities in the availability of psychosocial team members [10,11]. Existing team members are expected to fill the gaps [11,12].

In a report by the Canadian Society of Palliative Care Physicians Human Resources Committee [13] it was revealed that psychosocial support is lacking in Canadian palliative care. Only four of thirteen provinces and territories had interdisciplinary teams. Within these teams, psychology was reported by only 13.3% of respondents who worked in the teams, social work by 67.4%, and spiritual care by 52.3%. Psychiatrists were not differentiated from physicians and so their availability was not quantified. These numbers may suggest that psychosocial intervention is lacking in care or, alternatively, it might suggest that this proportion of psychosocial integration is meeting demand. We sought to further understand the interdisciplinary experience of Canadian palliative care professionals given these disparate gaps in care. In the current study, we examined reports of interdisciplinary integration as well as reports of what palliative professionals consider important interdisciplinary omissions in existing care.

## 2. Materials and Methods

### 2.1. Participants

This study was approved by the Health Research Ethics Board in Alberta Health Services (#HREBA.CC-17-0271). Participants were directors of palliative care programs in Canadian hospitals and universities, members of the Canadian Society for Palliative Care Physicians (CSPCP), and their interdisciplinary colleagues.

### 2.2. Materials/Interview Guide

Potential participants received an invitation to participate alongside an 11 item questionnaire assessing current practices in Canadian palliative care. In this report, we focus on open-ended data from question four: “Do you have multi-or interdisciplinary collaboration in your region (to the best of your ability, please comment on your local area and province)? Which disciplines are represented in your palliative care services”. In addition, we also included question five: “If there is multi- or interdisciplinary collaboration in your region’s palliative care services, how does the integration function in your region?” The reported demographics were also a focus of interest. There were two waves of recruitment (see Procedure, below). Materials were exclusively sent in English in Wave 1 of recruitment and French versions were added in Wave 2.

### 2.3. Procedure

Wave 1. We recruited a purposive sample of directors in palliative care from Canadian hospitals and universities. We identified directors by systematically searching the websites of hospitals and universities in each Canadian province and territory. The resulting list of institutions was crosschecked with our consultation team (authors AS and SC) and directors from missing institutions were further sought. We were unable to identify contacts in Saskatchewan, Yukon, North West Territories, and Nunavut.

Identified directors were emailed our invitation to participate, our questionnaire, and a request to forward our invitation to interdisciplinary colleagues (chain sampling). The interview was formatted as a Microsoft Word document for ease of reading and participants could respond directly in the document. If interested in participating, they could respond on the questionnaire directly or reply to arrange a phone interview with the research assistant/author MR.

We considered that eligible participants may also be colleagues of our research team and therefore may decline due to lack of confidentiality. To address this, our invitation email indicated that all communication would be conducted with our team’s research assistants (authors MR and MQ). It was disclosed that all raw data would be de-identified before sharing it with the larger research team.

Email invitations to participate in the research were sent on three occasions to potential participants, with two-week intervals between invitations to those who had not yet responded. After no response to three invitations, we considered them to have declined. Using this recruitment strategy, themes in our data did not reach saturation. In response, we added Wave 2.

Wave 2. We further recruited from a wide pool of palliative care physicians: members of the CSPCP. We received approval from the CSPCP to recruit through their mailing list. We sent out the same invitation, questionnaire, and request for chain sampling to their members who had agreed to receive such communications. We also added a French version of these documents to bolster the likelihood of response. CSPCP regulations allowed only one mail-out without repeated follow-up. They were unable to indicate the number of members who would have received this invitation.

### 2.4. Descriptive Characteristics

Research assistants de-identified each transcript and assigned ID numbers. We used a realist approach where the data are taken at face value. We operationalized reports of interdisciplinary professions as either “present” or “under/not-represented.” We qualified a reported profession as “present” if the participants indicated words such as available, core, daily, incorporated, or constant. We qualified a reported profession as “under/not-represented” if the participant used words such as missing, needed, desperate, requested, unavailable, irregular, or infrequent. We categorized the following as psychosocial clinicians: psychologists, social workers, psychiatrists, and spiritual care. We created two comparison groups from other professions reported by participants: medical (physicians except psychiatrists and nurses) and other (occupational therapists, physical therapist, dieticians, and volunteers).

### 2.5. Qualitative Data Analysis

We followed Vaismoradi, Turunen, and Bondas’ [14] procedure for content analysis to analyze clinician’s qualitative responses for descriptive information. Content analysis allowed us to consider both the relevance of clinicians’ responses when coding and also the frequency of their responses. A deductive approach to content analysis was undertaken. Transcripts were analyzed by using the a priori categories of the following: (a) what resources have clinicians said they have access to and (b) what resources do clinicians say they require more of. Our analysis procedure included developing familiarity with the transcripts through re-reading clinicians’ accounts. Open coding was completed based on directly observable content to code data from all clinicians into the two a-priori categories. And lastly, transforming the codes within each category into segments that could be discussed and supported by quotes. Discrepancies were resolved through peer debriefing (authors MR, MQ, and AF).

### 2.6. Rigor

While the quality of quantitative research is measured through reliability and validity, qualitative research often relies on markers of dependability, credibility, and transferability in order to establish rigor and trustworthiness of results [15,16]. Dependability was ensured by clearly articulating our methodological processes and referencing sources relevant to our research design. Credibility involves the fit between what the researcher produces and what the participants said. Credibility asks the researcher to be aware of the interpretation that comes from them and the interpretations that originate from the participants. This was ensured by prolonged engagement with the data and peer debriefing (e.g., discussing fit of transcripts with emerging codes and categories, alternate conceptualizations, methodological decisions, etc.). Lastly, whereas the outcome of quantitative research is generalizability, qualitative research leans towards transferability. Transferability of findings from qualitative research is decided on a case-by-case basis by the reader who must determine if their circumstance is similar enough to our participants to warrant transferability. We ensured this by providing as much details about participant demographics as we were ethically able to.

## 3. Results

Fourteen participants completed the survey (Wave 1: *n* = 13, wave 2: *n* = 1; see Table 1). Two participants chose a phone interview and 12 provided written responses. Response rates were the following: Ontario (*n* = 5); British Columbia, Alberta, Quebec, and Nova Scotia (*n* = 2 each); New Brunswick (*n* = 1). No responses were received from Manitoba, Prince Edward Island, and Newfoundland. We received no replies from Saskatchewan, Yukon, North West Territories, or Nunavut because there were no identifiable contacts for recruitment. Participants were physicians (*n* = 12), a nurse (*n* = 1), a social worker (*n* = 1), and an administrator (nurse by profession, *n* = 1). Participants worked in a combination of cancer care (*n* = 2), at a provincial cancer care agency (*n* = 1), mixed urban/suburban/and rural settings (*n* = 1), home care (*n* = 2), long-term care (*n* = 1), adult hospital care (*n* = 4), palliative care unit (*n* = 1), hospice (*n* = 3), pediatric hospital (*n* = 3), pediatric hospice (*n* = 3), academia (*n* = 1), management (*n* = 1), and an unstated location (*n* = 1). They could indicate multiple settings.

The most represented professions were the medical professions (physicians and nurses, *n* = 14) (see Table 2). Medical professionals were not reported by any participants as under/not-represented (*n* = 0). Psychologists were reported as “present” by only one participant (*n* = 1) and under/ not-represented by five participants (*n* = 5). Social workers were reported as represented (*n* = 7) and reported as under/not-represented (*n* = 5). Spiritual care was scarcely reported in both of the categories (represented *n* = 1 and under/not-represented *n* = 2). Other professions (occupational therapists, physiotherapists, dieticians, and volunteers) were reported as present (*n* = 10) and under/not-represented (*n* = 3). Psychiatrists were not reported at all in either category.

### 3.1. Qualitative Themes

#### 3.1.1. Commonly Represented Disciplines

When asked about interdisciplinary collaboration, participants mentioned a variety of professionals including: nurses, doctors, social workers, occupational therapists, physiotherapists, rehabilitation therapists, spiritual care, grief/bereavement support, child life specialists, dieticians, pharmacists, psychologists, and volunteers. There were 11 out of 14 participants who mentioned having access to or working with a combination of the above professions in their practice. There were nine who mentioned having representation of doctors, nurses, and social workers, whereas representation of other professionals varied more widely. One participant who was based in a hospice also mentioned having an ethicist, house cook, music therapist, and massage therapist. Another participant specifically mentioned collaborating with home care services and primary care teams in the community.

#### 3.1.2. Quality of Multidisciplinary Collaboration

Some respondents described that they had interdisciplinary collaboration in their hospital or that they felt their province was “good at ensuring interprofessional involvement.” Others elaborated more on how they felt about multidisciplinary collaboration in their province or region. For example, one participant qualified that they had “good interdisciplinary collaboration between their multidisciplinary team and many other multidisciplinary teams.” Another participant specified that they were based in a hospital and had good collaboration with their local hospice (for grief and bereavement support), continuing care/homecare, primary healthcare clinics, and volunteer coordinators. Another participant based in a hospice described that they had a strong core team of participants who were regularly involved and others who were invited on a “need to collaborate basis”, which worked well for them. While these three participants provided more positive accounts of collaboration, others mentioned that although they had multidisciplinary collaboration, it was “variable and location-dependent, not dedicated to palliative care” or that “disciplines work within the teams they are assigned to but not across services.”

#### 3.1.3. Commonly Under-Represented Disciplines

Despite the fact that 9 out of 14 participants mentioned having, at the minimum, doctors, nurses, and social workers on their teams and at times a variety of other professionals, 7 out of 14 participants had serious concerns about missing services and unmet needs. Participants’ most often repeated and most richly discussed need was for more social workers and psychologists. Other concerns included the following: the need for palliative specialist home-care services for patients in the community (*n* = 1), the need for adequate palliative care coverage of large regions (*n* = 1), and the fact that in some areas there is no regional palliative care service (*n* = 1).

#### 3.1.4. Special Concern: Psychosocial Care

Owing to the depth of participants’ responses about lack of psychosocial care, we discuss these findings in more detail here. The four participants who expressed concerns hailed from various disciplines and various provinces in the country but all had similar concerns—the lack of psychosocial resources for patients needing palliative care, specifically, through social work and psychology. As one participant put it, “there are major limitations on adequate physician/nursing resources; however, the constraints on psychosocial resources (social work, chaplaincy, psychology, child life, bereavement specialists) are so severe that they are almost non-existent.” Another participant expanded on this and described that their team used to have full time social work/grief support, a part-time chaplain, and a part time child life specialist who “met daily to discuss active patients (assessment, development of plan) and provided comprehensive care.” However, since program management moved away from the team approach, there has been less of an emphasis on team care and decreased involvement of social work and spiritual care. Lastly, one participant mentioned that at their palliative care facility they “do have physicians and nurses but are under-funded so lack in social workers/psychological help”, perhaps suggesting that when funding is short, these are the first disciplines to experience a cut.

## 4. Discussion

Our survey of palliative care clinicians and academics across Canada revealed that there remains a lack of psychosocial support in palliative care. This is despite the evidence that psychosocial distress decreases survival, quality-of-life, and heightens overall suffering [7,8,9]. Our Canadian findings replicate others [10,11,13] and also indicates that psychology and social work are the most noticed omission in palliative care.

Psychology and social work were both equally noted as under/not-represented (*n* = 5), however, only one participant reported presence of psychology versus seven who noted the presence of social work. The omission of psychology is consistent with the literature that integration in palliative care has been historically poor [10]. It also replicates the findings that hospital psychology is limited or omitted in Canadian medical care [17,18,19,20]. Our results highlight that despite widespread gaps and disparate care team formats, psychosocial are the most notable gaps amongst medical, psychosocial, and other disciplines.

For social work, our results revealed an interesting dichotomy. Social work was reported as the second most represented (second to medical) and tied as the most under/not-represented discipline. This may be understood as disparities across teams, indicating that the social worker may be the most likely psychosocial clinician if any are present [21]. Another interpretation is that without sufficient psychosocial staffing, the existing social workers may be spread too thin. This may limit them from practicing to scope or meeting needs of their patients/families, team members, workplace, or themselves.

Spiritual care was not mentioned frequently in either the represented (*n* = 1) or under/not-represented (*n* = 2) categories. This was in contrast to earlier findings that 52% of Canadian palliative care physicians reported spiritual care team members [13]. Their omission from current participants’ reports might indicate that (a) our participants were from a subset of the teams that possessed spiritual care (which is unlikely since not all of our participants had teams), (b) other team members are filling some of the gaps when spiritual care is not available (nurse, physicians, and volunteers), (c) our participants are not noticing this unmet need, and (d) spiritual care might be available through larger institutions and accidently omitted from reports. However, seven of our participants reported working in specialized palliative care or a hospice where the importance of spiritual care is usually understood. This needs to be investigated more.

Psychiatry was neither mentioned as represented or under/not-represented by participants. This may be because they were categorized with medical professionals and not uniquely named in any category. If they are also missing from care, it may be due to access limitations outside of urban centers or financial or human resource limitations. [22,23,24,25,26]. However, psychiatry trainees report strong interest in end-of-life care training [24,26]. In this regard, palliative care psychiatry with attention to psychosocial elements of end of life care is an emerging subspecialty [25]. 

Psychosocial clinicians were more often highlighted as under/not-represented than medical or other team members. This might indicate that there is a noticeable gap in care for participants. Other staff members may be able to address some less complex needs, although trained specialists are needed for the patients and families with higher degrees of distress [4,10,11,12,27]. Unaddressed emotional needs can cause excessive suffering for patients and families and the desire to mitigate this distress can result in alternate medical treatment decisions. It has been found that psychosocial suffering (e.g., hopelessness, feelings of burden, or social isolation) [27,28] is a strong contributor to the Desire for Hastened Death [27,29,30,31,32]. This is especially important when countries around the world are decriminalizing this practice.

Despite evidence for efficacy and importance of psychosocial interventions, Canadian palliative clinicians report that there is a lack of psychosocial presence, especially psychology and social work. There was nuance in the results in that participants’ answers varied widely with no discernable patterns based on their setting (e.g., hospice facility or hospital), location (e.g., province), and occupation (e.g., nurse and physician). Even within provinces, there was variation based on these individual differences of respondents, which perhaps speaks to the myriad of factors involved in trying to coordinating effective integrated palliative care and that each region may need its own specific solutions. However, as Hui et al. discusses, interdisciplinary teams create multidimensional care that address a patients’ physical, emotional, social, spiritual, and informational needs [33]. Without complete interdisciplinary teams, patients receive fragmented care.

We turned to our team of authors to speculate on barriers. Ideas included the following: At the department/institutional level, palliative care funding is usually allocated for medical management rather than mental health. Thus, it may be possible that hiring medical professionals such as nurses takes precedence over psychosocial care, especially if funding is limited. The consequences of this are that nurses and social workers are expected to fill the gaps of psychologists and spiritual care, but may need training to provide psychosocial support. This may also limit social workers in their ability to meet demands, which accounts for the ongoing reports of insufficient social workers in care. At a political/governmental level, the amount of funding available for healthcare and psychosocial care, in particular, depends on the priorities of political parties in power. At a sociocultural level, palliative care may not be popular training amongst particular psychosocial disciplines. North America has also been labeled as a “death denying” culture [13,34], perhaps rendering a field such as palliative care less appealing to mental health specialists. Finally, palliative care itself is under-represented in health care [13,34] and might not be programmatically in a position to support fully staffed teams.

A limitation to this study was that the data did not reach saturation despite a thorough recruitment strategy. The lack of rural hospitals and universities made it more difficult to reach the practitioners in rural areas. Our dataset was gleaned from a larger study and the current paper capitalized on the data we received. We may have been able to ask more directed questions or clarify our wonderings if our study had been focused on interdisciplinary team integration as a primary goal. Moreover, only two of our fourteen participants chose phone interviews rather than written responses, which limited our ability to probe for further information. The extent of transferability is determined by demographics of the sample (92.9% medical and 35.7% from Ontario). While valuable as a starting point, this is preliminary and more specific, targeted, and thorough research is needed.

## 5. Conclusions

There remains a stated need for psychosocial professionals fully integrated within palliative care across Canada. Reasons for these gaps might include lack of funding, distribution of funding, or the prioritization of physical symptoms over emotional symptoms. It is possible that teams are filling the gaps with other clinicians, which may meet some patient needs. However, clinicians are still reporting a need for specialized psychosocial professionals, especially psychologists and social workers, in this study.

Future research should investigate the barriers in hiring psychosocial professionals in palliative care. This may help us understand how Canadians can promote and advocate for these services. A targeted questionnaire may be considered for centers that are known to have a more integrated team of psychosocial clinicians to better understand how this can be achieved nationwide. Overall, identifying this gap in palliative care as well as the possible reasons for it will be the first step in providing patients and existing team members the support they need.

## Figures and Tables

**Table 1:** Participant response outcomes.

Recruitment	Number of Participants
Total invitation emails sent (wave 1 ^1^)	87
Completed participants (wave 1 and 2)	14
Incomplete/ unusable data	3
Total ineligible	0

^1^ Data unavailable for Wave 2.

**Table 2:** Reported professions.

Profession	Reported as Represented	Reported as Under/ Not-Represented
Medical (physicians and nurses)	14	0
Psychologists	1	5
Social workers	7	5
Spiritual Care/Chaplain	1	2
Psychiatrists	0	0
Other (occupational therapy, physical therapy, clinical nutrition, and volunteers)	10	3

## Data Availability

For the confidentiality of participants, qualitative raw data will not be made available.
